# Morphological and Structural Properties of Amino-Functionalized Fumed Nanosilica and Its Comparison with Nanoparticles Obtained by Modified Stöber Method

**DOI:** 10.3390/molecules25122868

**Published:** 2020-06-22

**Authors:** María C. Ruiz-Cañas, Laura M. Corredor, Henderson I. Quintero, Eduardo Manrique, Arnold R. Romero Bohórquez

**Affiliations:** 1 Grupo de Investigación en Química Estructural, Parque Tecnológico Guatiguará, Universidad Industrial de Santander, A.A. 678, Piedecuesta 681011, Colombia; 2 Instituto Colombiano del Petróleo, ECOPETROL S.A., A.A. 4185, Piedecuesta 681017, Colombia; laura.corredor@ecopetrol.com.co (L.M.C.); henderson.quintero@ecopetrol.com.co (H.I.Q.); eduardo.manrique@ecopetrol.com.co (E.M.)

**Keywords:** nanoparticles, surface activation, fumed silica, Stöber method, amino-functionalization

## Abstract

In industry, silica nanoparticles (NPs) are obtained by the fuming and the precipitation method. Fumed silica NPs are commonly used in the preparation of nanocomposites because they have an extremely low bulk density (160–190 kg/m^3^), large surface area (50–600 m^2^/g), and nonporous surface, which promotes strong physical contact between the NPs and the organic phase. Fumed silica has fewer silanol groups (Si–OH) on its surface than the silica prepared by the Stöber method. However, the number of –OH groups on the fumed silica surface can be increased by pretreating them with sodium hydroxide (NaOH) before further surface modification. In this study, the effectiveness of the NaOH pretreatment was evaluated on commercial fumed silica NPs with a surface area of 200 m^2^/g. The number of surface –OH groups was estimated by potentiometric titration. The pretreated fumed NPs, and the precipitated NPs (prepared by the Stöber method) were modified with 3-aminopropyltriethoxysilane (APTES) to obtain A200S and nSiO_2_-APTES, respectively. The NPs were characterized using electron dispersive scanning (EDS), scanning electron microscopy (SEM), dynamic light scattering (DLS), Fourier transform infrared spectroscopy (FT-IR), thermogravimetric analysis (TGA), X-ray diffraction (XRD), BET (Brunauer–Emmett–Teller) analysis, and ζ-potential. XRD confirmed the presence of the organo-functional group on the surface of both NPs. After the amino-functionalization, the ζ-potential values of the nSiO_2_ and A200 changed from −35.5 mV and −14.4 mV to +26.2 mV and +11.76 mV, respectively. Consequently, we have successfully synthesized functionalized NPs with interesting, specific surface area and porosity (pore volume and size), which can be attractive materials for chemical and energy industries.

## 1. Introduction

Research in nanotechnology over the past few years has resulted in different potential application areas for nanoparticles (NPs) [1,2,3,4,5,6]. The surface functionalization of NPs is often a precondition for applications where the interactions between components affect the stability of NPs or colloids [7,8]. The functionalization of NPs consists of the incorporation of reactive groups on the NPs surface [9,10,11,12,13], which become active sites to bind different molecules (i.e., polymers, composite materials, and elastomers) [14,15,16,17,18,19]. 

Different organic and inorganic compounds have been used to modify the surface of metal oxide NPs, including carboxylic acids [20,21], polymers [22,23,24,25,26], silanes [27,28,29], and organophosphorus molecules [12]. However, the silylation method using alkoxysilanes is the most commonly used because it leads to a much stronger interaction between the inorganic and organic phases of a nanohybrid or a nanocomposite [30]. Moreover, the silylation method may improve the stability of the NPs in an organic solvent or an aqueous solution by changing the surface charge or the nature of the NPs (from hydrophilic to hydrophobic) [31,32]. 

**Figure 1:**
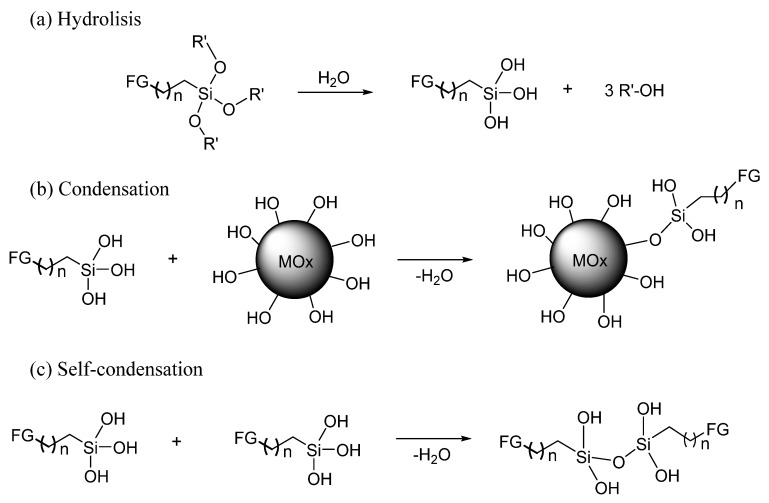
Scheme of the modification of metal oxide particles with an alkoxysilane derivative. (**a**) Hydrolisis; (**b**) Condensation; (**c**) Self-condensation. MOx: metal oxide, and FG: H, Hal-, NH_2_, SH, acryloxy group, between others.

Silane coupling agents have a nonhydrolyzable organic group (R–Y), which is attached to the surface of the metal oxide NPs and acts as a reactive or a hydrophobic site. It also contains hydrolyzable groups on the silicon atom. The hydrolysable groups are typically alkoxy (i.e., methoxy, ethoxy) [33,34] or chlorine groups [35,36]. The modification of the NPs surface with alkoxysilanes comprises two steps. First, the hydrolysis of the coupling agent produces reactive organosilanols (RSi-OH) (Figure 1a) and alcohol as a byproduct. Second, the condensation of the organosilanols with the hydroxyl groups on the NPs surface (via Si–O–M covalent bond) occurs to give organofunctional (Figure 1b). The self-condensation of the silane coupling agent can also take place in this step (Figure 1c). Organofunctional groups include amine [33,34,37], acryloxy [38,39,40], epoxy [41], isocyanate [22], and thiol [42] groups. If the organofunctional group is reactive, it can be covalently attached to a polymer matrix through radical chain transfer, addition, and cyclization reactions. For non-reactive groups, the silane can only improve the compatibility with the polymer matrix due to their similar polarities [43]. For practical purposes, the conditions of functionalization, modification, and preparation of desirable materials should be carefully optimized.

Silica NPs are produced by two methods: fuming and precipitation. Fumed silica is synthesized by the pyrolysis of silicon tetrachloride (SiCl_4_) in a flame of oxygen–hydrogen (Equation (1)). Precipitated silica is produced by precipitation from silicates and inorganic acids (sol-gel process) [12]. When the sol-gel reaction occurs under alkaline conditions, this is referred to as the Stöber method. Fumed silica has fewer silanol groups (Si–OH) on its surface than the precipitated one because it is produced at high temperatures (>1500 °C). For this reason, fumed silica NPs must be pretreated with sodium hydroxide (NaOH) before any surface modification.

(1) $$2H_{2}+O_{2}+{\;\mathrm{SiCl}}_{4}\;\underset{∆}{\to }{\;\mathrm{SiO}}_{2}+4\mathrm{HCl}$$

This work aimed to determine the effectiveness of the NaOH treatment on the preparation of amino-functionalized silica NPs. The NaOH treatment was used to activate the surface of commercial fumed SiO_2_ NPs with a surface area of 200 m^2^/g. After the pretreatment, the fumed and precipitated silica NPs (prepared by the Stöber method) were modified with 3-aminopropyltriethoxysilane (APTES). As stated in the literature, the APTES-modified silica NPs can be used in different fields, such as the preparation of hybrid materials [44,45,46], medicine [47,48,49,50], and many others [37,51,52]. Additionally, the APTES-SiO_2_ NPs studied in this work were successfully used to synthesize a SiO_2_-polyacrylamide (PAM) nanohybrid. The obtained nanohybrid exhibited better resistance to thermal degradation than the PAM [53].

## 2. Materials and Methods

### 2.1. Materials

Fumed silica NPs (A200) with a surface area of 200 m^2^/g were supplied by Evonik Industries AG (Essen, Germany). Tetraethyl orthosilicate (TEOS, 98%, Sigma-Aldrich, Louis, MO, USA), ethanol (EtOH, 96%, Merck Millipore, Burlington, MA, USA), and ammonium hydroxide (28−30 wt% solutions of NH_3_ in water, Merck Millipore, Burlington, MA, USA) were used to synthesize the silica NPs by the Stöber method. The activation reaction was carried out with sodium hydroxide (NaOH, 97%, Merck, Darmstadt, Germany), methanol (CH_3_OH, 99.9%, Merck, Germany), and acetic acid (C_2_H_4_O_2_, 100%, Merck, Darmstadt, Germany). Further, 3-aminopropyltriethoxysilane (APTES, 99%, Sigma-Aldrich, St. Louis, MO, USA) was used to functionalize the surface of the activated NPs. 

### 2.2. Nanoparticle Synthesis

The silica NPs were synthesized by following the method described by Stöber and Fink [54,55]. For this procedure, 1 mL (4.48 mmol) of TEOS was added in 32.5 mL of an ammonium hydroxide/ethanol solution (1:25 ratio). This dispersion (pH 10) was stirred for 3 h at 90 °C. Then, the silica NPs were centrifugally separated from the dispersion and washed three times with deionized water. Finally, the NPs were dried in a conventional oven for 24 h at 90 °C and they were named nSiO_2_. 

### 2.3. Activation Reaction of the Silica NPs

**Figure 2:**
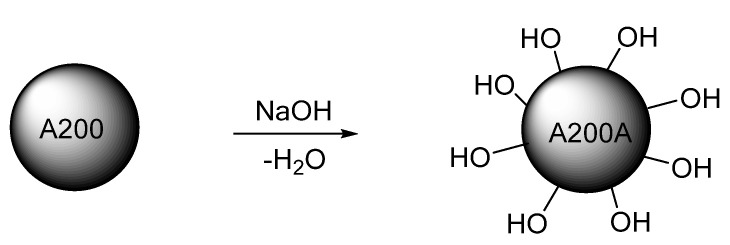
Activation of the fumed silica NPs.

The fumed silica NPs (A200) were activated by modifying their surface with hydroxyl ions (–OH) (Figure 2). For this purpose, 4 g of fumed silica NPs were dispersed in 100 mL of a 1 M aqueous solution of NaOH. The dispersion was stirred using a magnetic stirrer in a water bath at a temperature between 60 and 70 °C for 24 h. The dispersing media was neutralized with acetic acid, and the activated silica NPs were recovered by centrifugation. The powder was washed three times with methanol and dried for 24 h at 120 °C. This procedure was performed based on the method proposed by Lin et al. [56]. The obtained NPs were named A200A.

### 2.4. Amino-Functionalization Reaction

**Figure 3:**
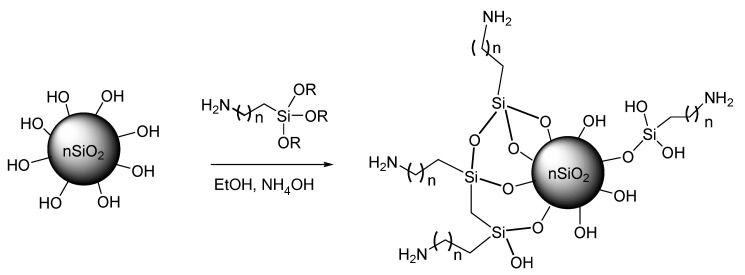
Amino-functionalization of the silica NPs.

All silica NPs were functionalized with 3-aminopropyltriethoxysilane (APTES) (Figure 3), generating a chemically reactive system. This procedure was performed based on the method proposed by Chen et al. [57]. In this procedure, 4 g of silica NPs were dispersed in a solution containing 2 g of APTES and 100 mL of ethanol. The dispersion was stirred for 3 h at room temperature. After treatment, the NPs were separated by centrifugation and alternately washed with ethanol and water at least two times to remove excess reagents. Finally, the NPs were dried for 12 h at 60 °C to evaporate volatile solvents. The silanized fumed and nSiO_2_ NPs were named A200S and nSiO_2_-APTES, respectively

### 2.5. Determination of the Hydroxyl Group Content on the NPs Surface

The content of –OH groups on the surface of the NPs was estimated by using the titration method described by Kang et al. [58]. In this method, 2 g of treated silica NPs were added into a bottle containing 80 mL of a 0.05 N aqueous solution of NaOH. The bottle was capped and stirred using a magnetic stirrer for 24 h at room temperature. The NPs were separated by centrifugation, and 10 mL of the supernatant were taken for titration. The indicator was prepared by adding 0.5 g of phenolphthalein into 100 mL of a mixture of water and ethanol (50% *v*/*v*). Then, three drops of the phenolphthalein solution were added into 10 mL of the supernatant. This sample was titrated until neutralization (when the color of the solution changed from magenta to transparent) with a 0.05 N aqueous solution of HCl. The same procedure was carried out for a blank solution (0.05 N aqueous solution of NaOH without silica NPs). For determination of the hydroxyl group content on the NPs surface, the corresponding Equation (2) was employed. Each titration was repeated at least three times to confirm the reproducibility, with the uncertainty found to be of the order of 0.01 mM/g of the reported value.

(2) $$X=\frac{\left(B-A\right)\times 0.05\times C}{W}$$

where *X* is the amount of -OH groups per unit weight of silica NPs (mM/g), *A* is the volume of HCl used to neutralize the sample containing NPs (mL), *B* is the volume of HCl used to neutralize the blank solution (mL). *W* is the amount of silica NPs used for the test and *C* is the ratio between the total volume of the solution (80 mL) and the aliquot volume (10 mL). 

### 2.6. NPs Characterization

A field emission gun scanning electron microscope (FEG-SEM) operating at an acceleration voltage of 25 kV (FEGSEM, QUANTA FEG 650 model, Thermo-Fisher Scientific, Waltham, MA, USA) was used to determine the size and morphology of the NPs. Each sample was placed on metal stubs with carbon adhesive tape and coated with gold using a Quorum 150 ES coating equipment (Quorum Technologies Ltd., Lewes, UK). The FEG-SEM images were obtained from the Everhart Thornley (ETD) and the backscattering electron detectors. The elemental composition of the NPs was determined by using an energy-dispersive spectroscope (EDS, EDAX Apolo X, Ametek, Inc., Berwyn, PA, USA) operating at an acceleration voltage of 25 kV.

The degree of crystallinity of the NPs was determined by X-ray diffraction at 2θ from 2° to 70° (Bruker D-8 A25 DaVinci, Bruker, Billerica, MA, USA). The patterns were recorded by a CuKα radiation source and a lynxEye detector at 0.6 mm divergence slit, 2.5° primary and secondary Soller slits, 40 kV accelerating voltage, and 40 mA current. The samples were loaded on polymethyl methacrylate (PMMA) holders with a Si center using the zero-background technique. 

Attenuated total reflection Fourier transform infrared spectroscopy (ATR-IR-FT) using a Bruker Tensor 27 FTIR spectrometer with a platinum cell (Alpha, Bruker, Billerica, MA, USA) was used to perform the structural characterization of all NPs. The FTIR spectra were recorded from 600 to 4000 cm^−1^ with a resolution of 4 cm^−1^ (24 scans were taken every 30 s for each spectrum).

The ζ-potential measurements were performed by using a Zetasizer ZS90 (Malvern Instruments, Herrenberg, Germany) equipped with a high concentration flow cell. Then, 100 ppm of NPs were dispersed in distilled water and sonicated for 15 min before measurement. The average ζ-potential value was calculated from three sets of measurements analyzed by the Zetasizer software version 7.13, where each measure was the mean of 50 individual runs carried out at different positions (10 measurements in 5 positions) in the flow cell. This analysis had an uncertainty of 0.5 mV at 25 °C. 

The hydrodynamic ratio of the NPs was determined by dynamic light scattering (DLS) analysis using a Zetasizer Nano ZS90 equipped with a 633 nm He-Ne laser (Malvern Instruments, Herrenberg, Germany) at an angle of 90° and 30 °C. For this test, 100 ppm of NPs were dispersed in deionized (DI) water. The dispersions were analyzed in a glass cell with a path length of 10 mm. The measurements were made at a 4.65 mm position from the cuvette wall using an automatic attenuator and equilibrium time of 180 s. Fifteen runs of 10 s were performed with three repetitions for each sample. The uncertainty in DLS results is 5.8 nm of the reported value. The software used to collect and analyze the data was the Zetasizer software version 7.13. The size distribution as a function of the intensity, the average diameter Z (Z-average), and the polydispersity index (PdI) were obtained from the autocorrelation function using the “general-purpose mode” for the monodispersed NPs. 

A fundamental parameter in the characterization of porous solids is their specific surface area. These measurements were conducted by using an ASAP 2020 specific surface analyzer (Micromeritics, GA, USA). Before each test, the sample was vacuum degassed at 398.15 K for 6 h. The surface area was measured by the low temperature (77 K) adsorption of nitrogen and calculated by the Brunauer, Emmett, and Teller (BET) method. The pore size distribution was calculated using the Barrett–Joyner–Halenda (BJH) method. The pore volume measurement allows estimating the degree of functionalization on the surface of the pores by comparing the pore volume of the functionalized with the non-functionalized NPs [59].

The thermal properties of the NPs were analyzed by thermogravimetry using a TA2050 TGA analyzer (TA Instruments, INC., New Castle, DE, USA). For the measurements, a mass of 5 mg of sample was heated from 25 to 800 °C at a nitrogen flow of 25 mL/min and a heating rate of 10 °C/min. Thermogravimetric analysis (TGA) allows for the determination of the organic matter content and the degree of functionalization in the NPs [60]. 

## 3. Results and Discussion

### 3.1. Determination of the –OH Groups Content on the NPs Surface

The content of the –OH groups on the NPs surface (A200, A200A, and nSiO_2_) was quantified by potentiometric titration and calculated using Equation (2). The content of –OH groups calculated was 0.07 mM/g, 1.90 mM/g, and 2.3 mM/g for A200, A200A, and nSiO_2_ NPs, respectively. This result showed that NaOH activated the A200 NPs surface by increasing the –OH content on their surface by 96%. In addition, the content of –OH groups of the A200A NPs (1.90 mM/g) is higher than that previously reported in the literature (1.1 mM/g) [56].

### 3.2. SEM—EDS Results

**Figure 4:**
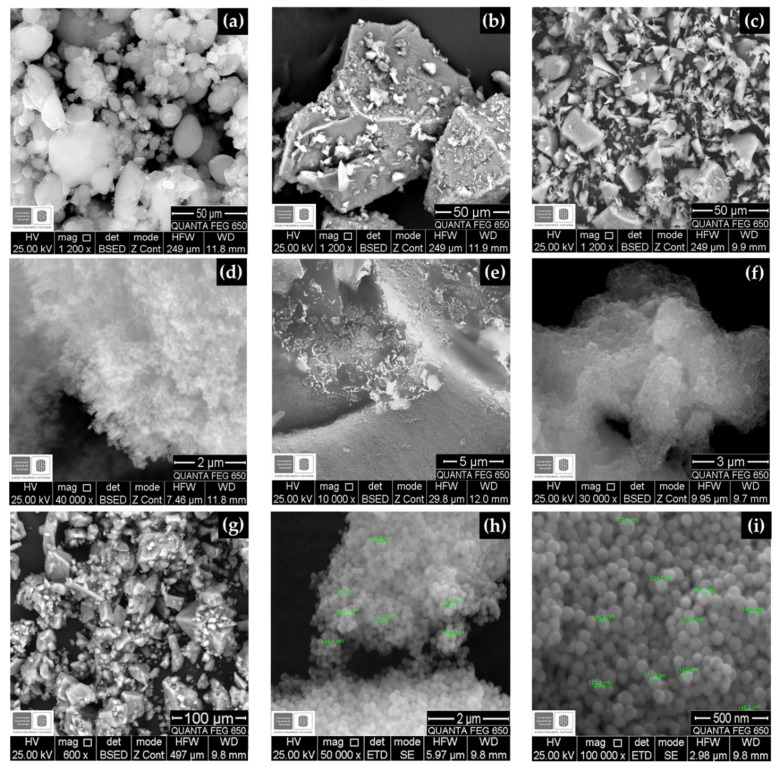
SEM micrographs of (**a**,**d**) A200 NPs at 1200× and 40,000×, respectively; (**b**,**e**) A200A NPs at 1200× and 10,000×, (**c**,**f**) A200S NPs at 1200× and 30,000× and (**g**–**i**) nSiO_2_-APTES NPs at 600×, 50,000× and 100,000×, respectively.

Figure 4 shows the micrographs of A200, A200A, A200S, and nSiO_2_-APTES NPs. The A200 NPs aggregated forming microspheres (Figure 4a,d) to reduce their high surface energy, resulting from their large surface-volume ratio. Moreover, the pretreatment with NaOH promoted the formation of aggregates, mainly as micron-size granules, due to the hydrogen bonding between the silanols on the surface (Figure 4b,e). The A200S NPs (Figure 4c,f) has an amorphous morphology and an average size of 60 nm. The nSiO_2_ –APTES have a near-spherical morphology and average size of 141 nm (Figure 4g–i) [53]. 

**Figure 5:**
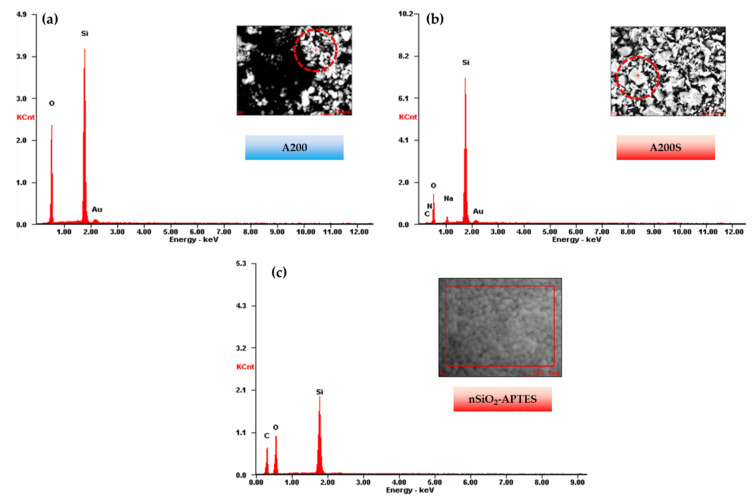
EDS results of (**a**) A200, (**b**) A200S and (**c**) nSiO_2_-APTES NPs.

Figure 5 shows the EDS results of A200, A200S, and nSiO_2_-APTES. The presence of C and N on A200S NPs confirms the attachment of the APTES onto the NPs surface (Table 1).

The relative oxygen content on the surface of A200S NPs decreased from 53.80 wt% to 32.69 wt% as compared with the A200 NPs (Table 1). In addition, the functionalization of the NPs is confirmed with the presence of C and N in the A200S and nSiO_2_-APTES NPs surface. The relative content of Si on the surface of nSiO_2_-APTES (22.20 wt%) was lower as compared with the A200S (53.31 wt%) due to the high surface area of A200 and the presence of silica in the structure

### 3.3. X-ray Diffraction (XRD)

**Figure 6:**
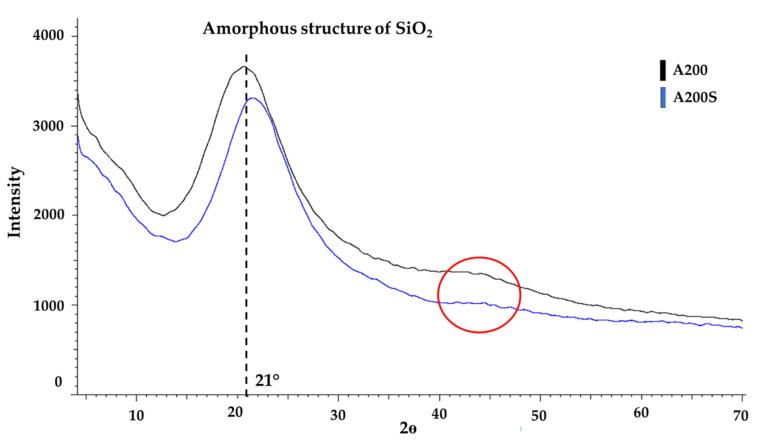
Diffractograms of A200 and A200S NPs.

XRD spectra of A200 and A200S NPs are shown in Figure 6. The spectra of A200 NPs exhibit a broad halo peak centered at a 2θ value of 21°, which confirms the amorphous structure of the NPs [11]_._ Also, the broad peak around 42° confirms the amorphous structure in the samples, which means that the unmodified NPs (A200) is highly disordered compared with the modified one (A200S) [61,62]. Upon the amino-functionalization, this signal was shifted to higher 2θ values. This was attributed to the attachment of the organic functional groups onto the surface of NPs channels, which tends to reduce the scattering power (or scattering contrast) of the amorphous silicate wall [63,64,65].

**Figure 7:**
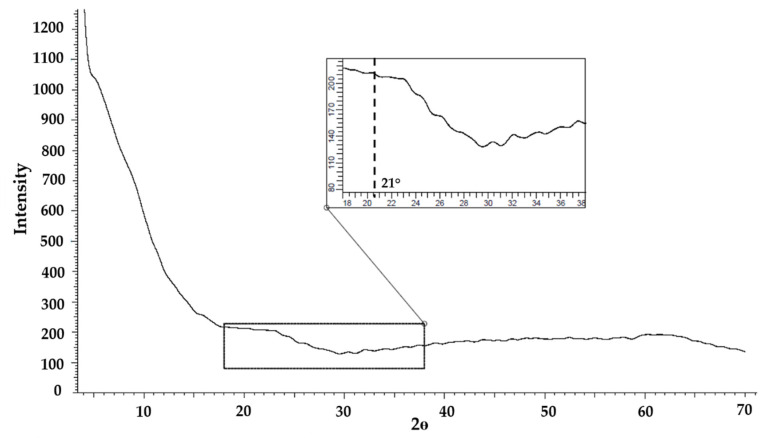
Diffractogram of nSiO_2_-APTES NPs.

Comparing A200S and nSiO_2_-APTES NPs (Figure 6 and Figure 7), a shift of the back of the main halo is observed (22° for A200S and 21° for nSiO_2_-APTES). This is attributed to an increment in the interplanar distance. The XRD results confirms that nSiO_2_-APTES NPs (spherical) have a different structure than the A200S NPs (amorphous).

### 3.4. FTIR-ATR Results

**Figure 8:**
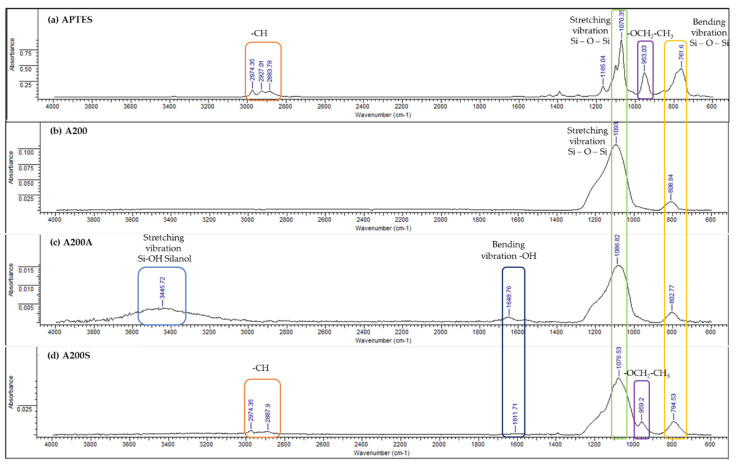
Infrared Spectrum of (**a**) APTES, (**b**) A200, (**c**) A200A and (**d**)A200S NPs.

Figure 8 displays the FTIR spectra of the A200, A200A, and A200S NPs. The absorption bands at around 1100 cm^−1^ and 809 cm^−1^ are attributed to the stretching and bending vibration of the siloxane groups (Si–O–Si), respectively [66,67]. Compared to the unmodified NPs, two new adsorption bands at 3446 cm^−1^ and 1649 cm^−1^ are observed on the IR spectrum of the A200A NPs (Figure 8b), which are assigned to the stretching vibration of the silanol group (Si–OH) [68,69] and adsorbed water in the sample [70], respectively. These results confirm the presence of hydroxyl groups on the surface of the NPs. The A200 modification by APTES is evidenced with the bands at 2974 cm^−1^, and 959 cm^−1^ (Figure 8c). These bands are attributed to C–H stretching vibration, and the unhydrolyzed ethoxy moieties in the APTES, respectively [32,71,72,73]. Furthermore, the disappearance of the band at 3446 cm^−1^ confirms the surface modification of the NPs with APTES.

**Figure 9:**
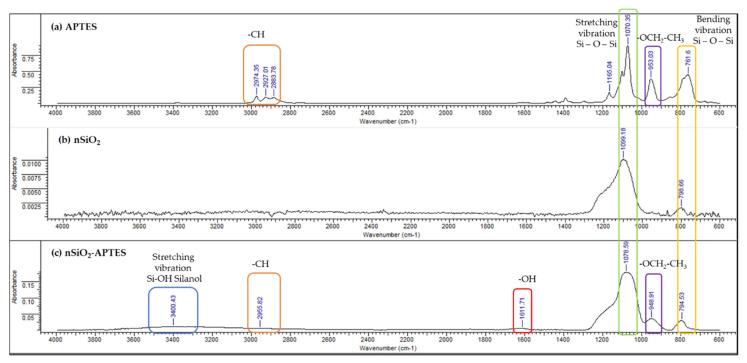
Infrared spectra of (**a**) APTES, (**b**) nSiO_2_ and (**c**) nSiO_2_-APTES NPs.

The FTIR spectra of APTES, n-SiO_2_, and n-SiO_2_-APTES (Figure 9) show typical bands with the spectra of the fumed NPs reported in Figure 8. The bands at around 1100 and 800 cm^−1^ are ascribed to asymmetric stretching and bending vibrations of the siloxane groups (Si–O–Si), respectively [69]. In the APTES spectra, the characteristic bands are 2974 cm^−1^ and 953.03 cm^−1^, which are assigned to C–H stretching vibration and –OCH_2_-CH_3_–vibration, respectively [32]. The SiO_2_-APTES NPs spectra show a new band at 949 cm^−1^, which is attributed to the unhydrolyzed ethoxy moieties in the APTES. These results confirm the presence of organic substitution in the modified silica [73].

### 3.5. Colloidal Stability (ζ-Potential)

**Figure 10:**
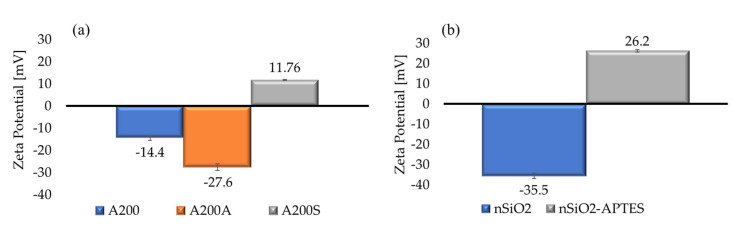
ζ-potential values of (**a**) A200, A200A and A200S, and (**b**) nSiO_2_ and nSiO_2_-APTES NPs.

The colloidal stability of NP dispersions is predicted by the magnitude of the ζ-potential. Dispersions with ζ-potential values greater than −30 mV or less than +30 mV are usually unstable due to interparticle attractions [74,75,76]. The ζ-potential values of all NPs dispersed in distilled water are presented in Figure 10. It is observed that only the n-SiO_2_ dispersion is stable, according to the DLVO theory. The higher degree of stability of nSiO_2_ dispersion, compared to the A200, is attributed to the dissociation of silanol groups on the NPs surface. The absolute ζ-potential value increase of the A200 NPs was consistently observed after the pretreatment with NaOH (from −14.4 to −27.6 mV). Upon the NPs amino-functionalization, the A200S and nSiO_2_-APTES potential changed to +11.76 mV and +26.2 mV, respectively. The latter can be directly related to the protonation of the amine group. This effect confirms that enough surface coverage with APTES was achieved. Both dispersions are unstable due to the hydrophobicity of the APTES grafted to the surface.

### 3.6. Hydrodynamic Radius by DLS Results

**Figure 11:**
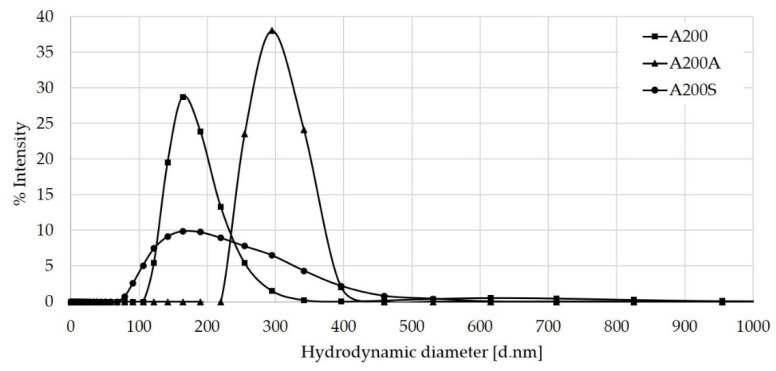
DLS results of A200, A200A, and A200S NPs.

**Figure 12:**
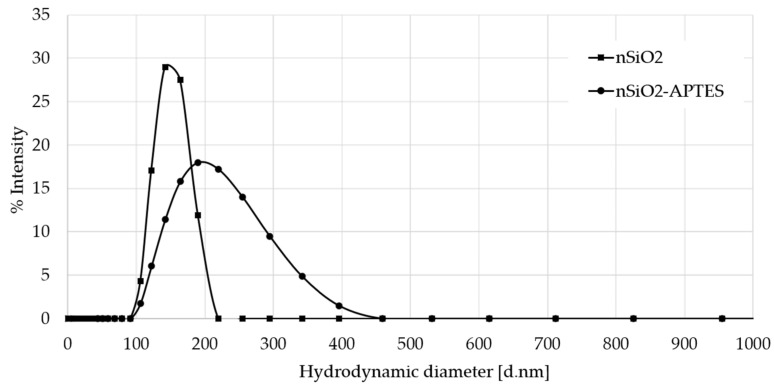
DLS results of nSiO_2_ and nSiO_2_-APTES NPs.

After the NaOH pretreatment, the average size of the A200 NPs increased from 164 to 295 nm (Figure 11). This increase is due to the NP aggregation through the hydrogen bonding between the silanols on the NPs surface. However, the increased stability of the NPs observed in the ζ-potential measurements suggests the formation of a hydration layer that stabilizes the aggregates. For A200S NPs, a broader particle size distribution is observed (high polydispersity). This is attributed to the formation of aggregates due to hydrophobic interactions between NPs, which is consistent with its low ζ-potential value. Similar behavior was observed for the nSiO_2_ and nSiO_2_–APTES NPs (Figure 12). However, these NPs showed a smaller hydrodynamic radius and less polydispersity than the fumed NPs. These characteristics make them more stable in aqueous solutions (higher absolute ζ-potential values). 

### 3.7. BET Isotherms

**Figure 13:**
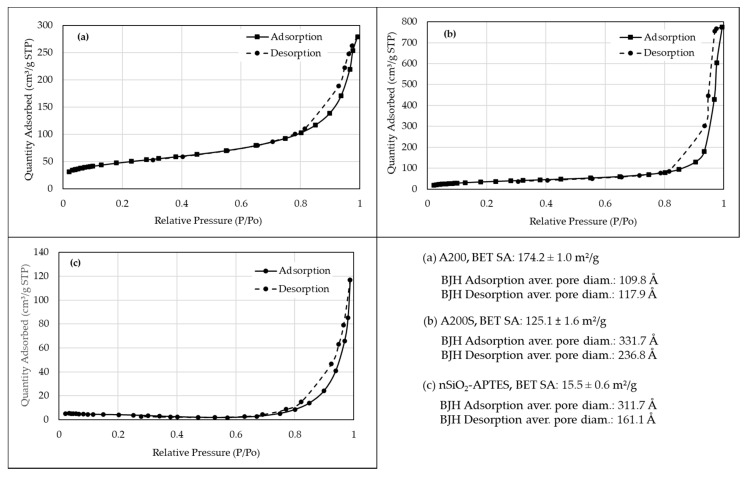
Sorption isotherms of N_2_ on (**a**) A200, (**b**) A200S and (**c**) nSiO_2_-APTES NPs at 77 K.

The specific surface area and pore size of all NPs were calculated from N_2_ adsorption/desorption isotherms (Figure 13). According to the IUPAC classification [77], all NPs exhibited a type IV adsorption isotherm with a type H1 hysteresis loop very narrow in the relative pressure range of 0.8–1.0. This type of isotherm is characteristic of mesoporous materials, and it is characterized by a hysteresis curve caused by capillary condensation in mesopores with open cylindrical cavities [78,79,80]. According to the results of the BHJ method, all NPs have a pore size in the mesoporous range, between 20 and 500 Å. 

The specific surface area of A200S NPs (125.13 ± 1.64 m^2^/g) is lower than that of the A200 NPs (174.18 ± 1.03 m^2^/g) due to the amino-functionalization of the NPs (Figure 13a,b). The surface area calculated for A200 NPs agrees with that reported by the manufacturer (Evonik) [81]. Additionally, the surface area of the A200S NPs (125.13 ± 1.64 m^2^/g) is higher than that of the nSiO_2_-APTES NPs (15.52 ± 0.63 m^2^/g) (Figure 13c) because the APTES can easily fill the uniform micropores and restrict the access of nitrogen into them [82].

### 3.8. TGA Results

**Figure 14:**
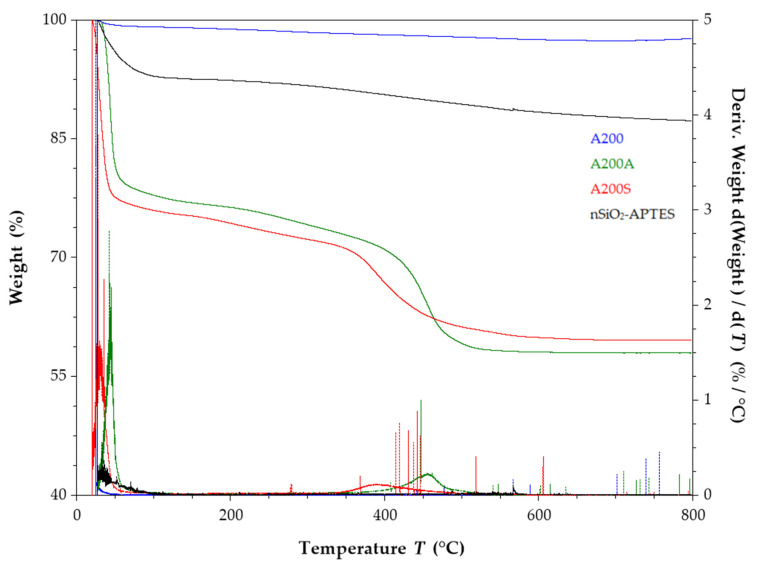
Thermograms of A200, A200A, A200S and nSiO_2_-APTES NPs (heating rate of 10 °C/min in a nitrogen atmosphere).

The unmodified A200 show a slight weight loss of 1.4% between 100–800 °C (Figure 14), which was attributed to the surface dehydration and dehydroxylation [37]. By comparison, the first weight loss of the A200S NPs (24.3%) occurred below 100 °C, and it was assigned to the surface dehydration. The weight loss at temperatures above 100 °C is divided into two regions. The first weight-loss region occurs in the 100–350 °C range, and it can be attributed to the condensation of silanol groups of aminopropyl groups, and between them and the residual surface silanols on the NPs surface. The weight loss between 350 and 600 °C can be attributed to the thermal decomposition of the aminopropyl groups [32,83]. This result shows that the temperature for the decomposition of the aminopropyl groups on the NPs surface is much higher than the boiling point of the APTES (217 °C). The latter indicates that the aminopropyl groups are covalently attached to the SiO_2_ NPs and have higher thermal stability than the modifier. No weight loss was observed above 600 °C.

On the other hand, the weight loss of nSiO_2_-APTES and A200S NPs was 2.8% and 11.2% in the range of 350 and 600 °C, respectively. The latter indicates that the precipitated NPs have a smaller extent of functionalization with APTES than the fumed NPs. This behavior was attributed to the structural difference between them.

## 4. Summary and Conclusions

In this study, the activation and amino-functionalization of commercial fumed and precipitated silica NPs prepared by the Stöber method were evaluated. The NPs were characterized through XRD, FTIR, TGA, BET, SEM, and DLS. Additionally, the colloidal stability of the NPs in distilled water was predicted by ζ-potential. The increase of the content of –OH groups on the A200 NPs surface by pretreatment with NaOH (1.90 mM/g) was confirmed by potentiometric titration. The amino-functionalization of the NPs was confirmed by FTIR analysis. The comparative study of SEM, XRD nitrogen adsorption, and DLS results of A200S and nSiO_2_-APTES NPs showed that the precipitated NPs have a spherical shape, lower surface area, and larger particle size than the fumed ones. Upon the amino-functionalization of the NPs, the sign of the ζ-potential values changed (from negative to positive). Still, the stability of the dispersions did not improve due to the hydrophobic interactions between NPs.

## Figures and Tables

**Table 1:** Weight and atomic percentage of A200, A200S and nSiO_2_-APTES S NPs by EDS analysis.

Element	A200		A200S		nSiO_2_-APTES	
	wt%	at%	wt%	at%	wt%	at%
C			3.44	6.35	36.29	47.16
N			1.17	1.85	0.50	0.50
O	53.80	65.16	32.69	45.35	41.51	40.50
Si	36.84	25.41	53.31	42.12	22.20	12.34
Na			3.83	3.70		
